# The impact of brain lesion characteristics and the corticospinal tract wiring on mirror movements in unilateral cerebral palsy

**DOI:** 10.1038/s41598-022-19920-z

**Published:** 2022-09-29

**Authors:** Cristina Simon-Martinez, Lisa Decraene, Ingar Zielinski, Brian Hoare, Jacqueline Williams, Lisa Mailleux, Bert Steenbergen, Els Ortibus, Hilde Feys, Katrijn Klingels

**Affiliations:** 1 Department of Rehabilitation Sciences, KU Leuven - University of Leuven, Leuven, Belgium; 2 Institute of Information Systems, University of Applied Sciences Western Switzerland (HES-SO) Valais, Rue de Techno-Pôle 3, 3960 Sierre, Switzerland; 3 Division of Neuropediatrics, Development and Rehabilitation, University Children’s Hospital, Inselspital, Bern University Hospital, University of Bern, Bern, Switzerland; 4 Rehabilitation Research Centre - REVAL, Faculty of Rehabilitation Sciences, Hasselt University, Diepenbeek, Belgium; 5 Behavioural Science Institute, Radboud University Nijmegen, Nijmegen, The Netherlands; 6 Clinic for Pediatric and Adolescent Psychiatry, Psychosomatics, and Psychotherapy, Uniklinik RWTH, Aachen, Germany; 7 School of Occupational Therapy, La Trobe University, Melbourne, VIC Australia; 8 Department of Paediatrics, Monash University, Melbourne, VIC Australia; 9 College of Sport and Exercise Science, Institute for Health and Sport, Victoria University, Melbourne, VIC Australia; 10 School of Psychology, Australian Catholic University, Melbourne, Australia; 11 Centre for Disability and Development Research, Australian Catholic University, Melbourne, Australia; 12 Department of Development and Regeneration, KU Leuven - University of Leuven, Leuven, Belgium

**Keywords:** Medical research, Outcomes research, Paediatric research

## Abstract

Mirror movements (MM) influence bimanual performance in children with unilateral cerebral palsy (uCP). Whilst MM are related to brain lesion characteristics and the corticospinal tract (CST) wiring pattern, the combined impact of these neurological factors remains unknown. Forty-nine children with uCP (mean age 10y6mo) performed a repetitive squeezing task to quantify similarity (MM-similarity) and strength (MM-intensity) of the MM activity. We used MRI data to evaluate lesion type (periventricular white matter, N = 30; cortico-subcortical, N = 19), extent of ipsilesional damage, presence of bilateral lesions, and damage to basal ganglia, thalamus and corpus callosum. The CST wiring was assessed with Transcranial Magnetic Stimulation (17 CSTcontralateral, 16 CSTipsilateral, 16 CSTbilateral). Data was analyzed with regression analyses. In the more-affected hand, MM-similarity and intensity were higher with CSTbilateral/ipsilateral. In the less-affected hand, MM-similarity was higher in children with (1) CSTcontra with CSC lesions, (2) CSTbilat/ipsi with PVL lesions and (3) CSTbilat/ipsi with unilateralized lesions. MM-intensity was higher with larger damage to the corpus callosum and unilateral lesions. A complex combination of neurological factors influences MM characteristics, and the mechanisms differ between hands.

## Introduction

One of the impairments that may hamper the performance of daily life activities in children with unilateral cerebral palsy (uCP) is the presence of mirror movements (MM)^1^. MM are described as involuntary movements in one limb, which are initiated by simultaneous, voluntary activation of the homologous muscles in the other limb^2,3^. MM are a physiological feature in typically developing children, gradually disappearing during the first decade of life^2^ due to the gradual maturation of descending tracts and the corpus callosum^4^.

In many children with uCP, MM are more pronounced and persistent^3,5,6^ and negatively impact the performance of asymmetric bimanual tasks and the time required to complete them^1^ as well as response to treatment^7^. Two hypotheses have been put forward as potential mechanisms of MM occurrence in uCP: (1) an ipsilateral corticospinal tract (CST) projecting from the contralesional motor cortex to the more affected hand; and (2) insufficient interhemispheric inhibition between the two primary motor cortices^8^. The reorganization of the CST in three wiring patterns (i.e. contralateral (CSTcontra), ipsilateral (CSTipsi) and bilateral (CSTbilat), is unique in children with uCP due to the underlying brain lesion and refers to the efferent motor input to the affected hand^9,10^. The presence of an ipsilateral tract (either in CSTipsi or CSTbilat wiring pattern) has been found to be related to more severe MM, even though children with CSTcontra may also present MM^11–14^. However, to the best of our knowledge, there are no studies investigating the impact of interhemhispheric inhibition on MM in children with uCP. In children with bilateral CP, a simultaneous bilateral activation of both primary motor cortices and insufficient interhemispheric inhibition, together with decreased fiber integrity in the transcallosal pathways has been related to MM^15^. However, this mechanism may vary between types of CP and between hands.

Brain lesion characteristics such as the lesion type, extent and location and the presence of bilateral lesions could provide additional information to the underlying mechanism of MM occurrence. The *type of lesion*, related to the timing of the lesion during gestation, can be classified into three categories: malformations (1st and 2nd trimesters of pregnancy), periventricular white matter lesion (PV, early 3rd trimester), and cortico-subcortical lesions (CSC, late 3rd trimester and around birth)^16^. Klingels et al.^6^ demonstrated that malformations and PV lesions were related to more severe MM in the more-affected hand than other lesion types. However, Riddell et al.^12^ found no difference between lesion groups in the more-affected hand. These contrasting results highlight the need for further investigation, including the influence of other brain lesion characteristics.

Additional studies have reported a relationship between *lesion extent* and MM occurrence. The extent of the lesion in children with PV lesions has been related to MM occurrence, whereby a larger lesion was related to stronger MM^17^. Whether this relationship also applies to other lesion types i.e. malformations and CSC, remains unknown. Other brain lesion characteristics influencing MM, such as *lesion location* and the presence of *bilateral lesions*, have not previously been investigated.

In previous studies, the use of an observation-based scale producing ordinal data (i.e. Woods and Teuber^5^) hampers objective quantification of the MM activity. In this study, quantitative methods with advanced analyses that better identify different MM characteristics^18^ may provide deeper insights in the MM phenomenon and the neurological factors influencing it. The aim of the study is to investigate the role of discrete neurological factors (i.e. brain lesion characteristics and CST wiring pattern), as well as their combined impact on MM characteristics in children with uCP, using a novel, objective method to quantify MM^18,19^. We hypothesize that a combination of neurological factors explains MM in each hand. More specifically, we hypothesize that greater similarity and strength of MM in the more-affected hand will be related to earlier brain lesions and will occur greatly in children with CSTipsi or CSTbilat^6,13,14^. As MM in the less-affected hand are not related to the type of CST wiring, we hypothesize that they may be related to the interhemispheric interactions (either facilitation or inhibition), which is linked to the damage to the corpus callosum and the lesion asymmetry. The identification of the combined impact of the neurological factors will aid in better explaining MM characteristics in children with uCP.

## Results

### Participants

Eighty-six children with uCP were recruited for this study (mean age 10 years 6 months, SD 2 years 6 months, 43 girls, 45 right-sided uCP). We only included participants with complete datasets (MM data, brain lesion characteristics and CST wiring pattern), and we excluded brain malformations due to low incidence^20^, resulting in a sample of 49 children (mean age 10 years 6 months, SD 2 years 8 months, 25 girls, 24 right uCP, MACS: 18 MACS I, 18 MACS II and 13 MACS III). Reasons for exclusion were either related to the MM evaluation (n = 2), TMS assessment (n = 20), MRI session (n = 11) or mixed reasons (n = 4) and are described in detail in Supplementary Materials Table S1. Thirty children had a PV and 19 a CSC lesion; 30 had a purely unilateral lesion and 19 had a bilateral brain damage. The CST wiring pattern was CSTcontra in 17 children, CSTipsi in 16 and CSTbilat in 16. Demographic and MM data did not differ between sites (Leuven, Belgium n = 44, Melbourne, Australia n = 5; Table 1).

**Table 1:** Differences between sites in demographic, neurological, and mirror movement data.

		Leuven (n = 44)	Melbourne (n = 5)	Between-group differences (p-value)	Total
**Age**	X (SD)	10.52 (2.63)	10.60 (3.73)	0.96	10.53 (2.71)
**Gender**					
Girl	n (%)	21 (47.7%)	4 (80%)	0.35	25 (51%)
Boy		23 (52.3%)	1 (20%)		24 (49%)
**More-affected upper limb**					
Left	n (%)	25 (56.8%)	0 (0%)	*0.02*	25 (51%)
Right		19 (43.2%)	5 (100%)		24 (49%)
**MACS**					
I	n (%)	17 (38.6%)	1 (20%)	0.12	18 (36.7%)
II		14 (31.8%)	4 (80%)		18 (36.7%)
III		13 (29.5%)	0 (0%)		13 (26.5%)
**CST wiring**					
Contralateral	n (%)	14 (31.8%)	3 (60%)	0.48	17 (34.7%)
Bilateral		15 (34.1%)	1 (20%)		16 (32.7%)
Ipsilateral		15 (34.1%)	1 (20%)		16 (32.7%)
**Lesion timing**					
PV	n (%)	27 (61.4%)	3 (60%)	1	30 (61.2%)
CSC		17 (38.6%)	2 (40%)		19 (38.8%)
**MM in more-affected hand**					
Similarity (CCC)	X (SD)	0.29 (0.27)	0.29 (0.16)	0.74	0.29 (0.26)
Intensity (force)		41.04 (35.94)	58.52 (52.1)	0.36	42.83 (37.58)
**MM in less-affected hand**					
Similarity (CCC)	X (SD)	0.32 (0.28)	0.43 (0.2)	0.27	0.33 (0.28)
Intensity (force)		38.94 (49.24)	33.4 (41.23)	0.95	38.38 (48.13)

MACS, manual ability classification system; CST, corticospinal tract; PV, periventricular; CSC, cortico-subcortical; MM, mirror movements; CCC, cross-correlation coefficient; X, mean; SD, standard deviation.

Significant value is in italic.

### Relation between MM and more-affected side, sex, age and hand function

MM characteristics did not differ according to more-affected side (MM-similarity, p > 0.67, r_rb_ = 0.07; MM-intensity, p > 0.33, r_rb_ = 0.04–0.17), sex (MM-similarity, p > 0.23, r_rb_ = 0.10–0.20; MM-intensity, p > 0.24, r_rb_ = 0.06–0.20) nor were related to age (MM-similarity, rho = 0.02 to 0.07, p > 0.54; MM-intensity, rho = − 0.02 to − 0.13, p > 0.36). MM characteristics differed according to manual ability levels (MM-similarity, χ^2^ = 8.98–14.24, p ≤ 0.01, ε^2^ = 0.30–0.19; MM-intensity, χ^2^ = 11.51–12.13, p < 0.005, ε^2^ = 0.24–0.25), whereby children at MACS level II showed more similar and stronger MM in the less-affected hand and children at MACS level III showed stronger MM in the more-affected hand (Table 2) compared to children at MACS level I. Additionally, worse unimanual capacity (Jebsen-Taylor Hand Function test) correlated lowly with stronger MM in both hands (rho = 0.37–0.58, p < 0.01) and to higher MM similarity in the less-affected hand (rho = 0.38, p = 0.01).

**Table 2:** Differences in MM characteristics according to manual ability (MACS levels).

		MACS I	MACS II	MACS III	p
**More-affected hand**					
MM-similarity	Me (IQR)	0.1 (0.0–0.3)	0.4 (0.2–0.7)	0.2 (0.1–0.3)	0.03^a^
MM-intensity	Me (IQR)	12.8 (7.0–17.6)	35.4 (16.7–74.8)	61.6 (43.2–78.5)	0.004^b^
**Less-affected hand**					
MM-similarity	Me (IQR)	0.1 (0.0–0.2)	0.5 (0.4–0.7)	0.3 (0.2–0.5)	< 0.001^a^
MM-intensity	Me (IQR)	6.7 (3.9–10.7)	42.2 (19.6–77.5)	21.6 (11.7–57.8)	0.006^a^

Me, median; IQR, interquartile range; MM, mirror movements; MACS, manual ability classification system.

^a^MACS I versus MACS II.

^b^MACS I versus MACS III.

### Influence of each neurological factor on mirror movements

Table 3 reports the MM data in each hand according to the CST wiring pattern and the brain lesion characteristics, as well as the association of each variable with the MM characteristics. Additionally, we report the relation between each neurological factor on mirror movements with the individual data points in Supplementary Materials (Suppl. Figure S1-S8).

**Table 3:** Descriptive statistics (X (SD)) and simple regression analyses of MM similarity and MM intensity according to the CST wiring and the brain lesion characteristics.

	More-affected hand						Less-affected hand					
	MM-similarity			MM-intensity			MM-similarity			MM-intensity		
	X (SD)	R^2^ (p-value)^a^	Std. Est. (p-value)^b^	X (SD)	R^2^ (p-value)^a^	Std. Est. (p-value)^b^	X (SD)	R^2^ (p-value)^a^	Std. Est. (p-value)^b^	X (SD)	R^2^ (p-value)^a^	Std. Est. (p-value)^b^
**CST wiring pattern**		0.26 (0.02)*			0.31 (0.006)*			0.43 (< 0.001)*			0.21 (0.07)	
Contralateral	0.18 (0.19)			24.85 (36.63)			0.16 (0.19)			18.04 (29.68)		
Bilateral	0.32 (0.26)		0.35 (0.34)	38.16 (22.99)		0.01 (0.98)	0.36 (0.25)		0.37 (0.24)	45.69 (57.55)		0.25 (0.50)
Ipsilateral	0.40 (0.29)		0.81 (0.03)*	66.59 (39.58)		0.70 (0.06)	0.49 (0.29)		0.94 (0.005)*	52.67 (49.16)		0.49 (0.20)
**Lesion type**		0.20 (0.04)*			0.22 (0.03)*			0.31 (0.002)*			0.25 (0.011)*	
Periventricular lesion	0.27 (0.26)			39.29 (39.51)			0.34 (0.31)			27.55 (37.32)		
Cortico-subcortical lesion	0.33 (0.26)		0.35 (0.22)	48.42 (34.59)		0.14 (0.62)	0.33 (0.23)		0.01 (0.97)	55.47 (58.57)		0.59 (0.04)*
**Lesion location**												
Frontal lobe		0.17 (0.08)	0.04 (0.79)		0.21 (0.03)*	0.03 (0.83)		0.35 (< 0.001)*	− 0.19 (0.13)		0.19 (0.06)	− 0.10 (0.47)
Parietal lobe		0.18 (0.07)	0.09 (0.55)		0.23 (0.02)*	− 0.11 (0.42)		0.34 (< 0.001)*	− 0.17 (0.20)		0.18 (0.11)	0.10 (0.49)
Basal Ganglia and Thalamus		0.20 (0.04)*	0.19 (0.18)		0.24 (0.01)*	0.18 (0.20)		0.31 (0.002)*	− 0.01 (0.92)		0.20 (0.04)*	0.18 (0.22)
Corpus callosum		0.20 (0.04)*	0.23 (0.13)		0.21 (0.03)*	0.06 (0.70)		0.34 (0.002)*	0.18 (0.18)		0.31 (0.003)*	0.39 (< 0.01)*
Bilaterality												
Unilateral	0.33 (0.27)	0.18 (0.06)		52.51 (41.01)	0.26 (0.009)*		0.42 (0.28)	0.39 (< 0.001)*		51.13 (55.95)	0.24 (0.02)*	
Bilateral	0.23 (0.23)		− 0.26 (0.37)	27.54 (25.56)		− 0.46 (0.10)	0.20 (0.22)		− 0.58 (0.03)*	18.24 (20.85)		− 0.52 (0.07)
Asymmetry Index		0.20 (0.04)*	− 0.20 (0.17)		0.24 (0.02)*	− 0.17 (0.24)		0.38 (< 0.001)*	− 0.29 (0.03)*		0.21 (0.03)	− 0.20 (0.17)
**Lesion extent**		0.20 (0.04)*	0.18 (0.21)		0.21 (0.03)*	0.03 (0.82)		0.31 (0.002)*	− 0.03 (0.81)		0.22 (0.03)*	0.22 (0.52)

MM, mirror movements; CST, corticospinal tract; X, mean; SD, standard deviation.

Significant values are in bold.

^a^Results of the full model controlling for hand function (MACS levels) and age (continuous variable).

^b^Standardized estimates and p-value of the variable of interest in the full corrected model. The categorical values are entered in the model as dummy variables, and the reference levels are contralateral CST, periventricular lesion and unilateral bilaterality. If the full model is statistically significant, it may be due to the influence of the covariates (hand function and age). The sign of the standardized estimates indicates whether the particular variable has a negative or positive influence on the independent variable (i.e., MM characteristic). For categorical variables (CST wiring pattern, lesion type and bilaterality), the first category (contralateral, PV lesion and unilateral) serves as baseline, and the other categories are compared to the first one.

All factors influenced MM characteristics (Table 3), although in a different way. When controlling for manual ability and age, the CST wiring pattern and the type of the lesion influenced both MM-similarity and MM-intensity in both hands, as did the damage to specific locations (basal ganglia and corpus callosum), having unilateral lesions and the extent of the lesion. Higher MM in both hands were present in children with CSTipsi, CSC lesions, larger damage to the basal ganglia and corpus callosum, a more lateralized lesion, and a larger lesion extent.

### Combined influence of the neurological factors on mirror movements

**Figure 1:**
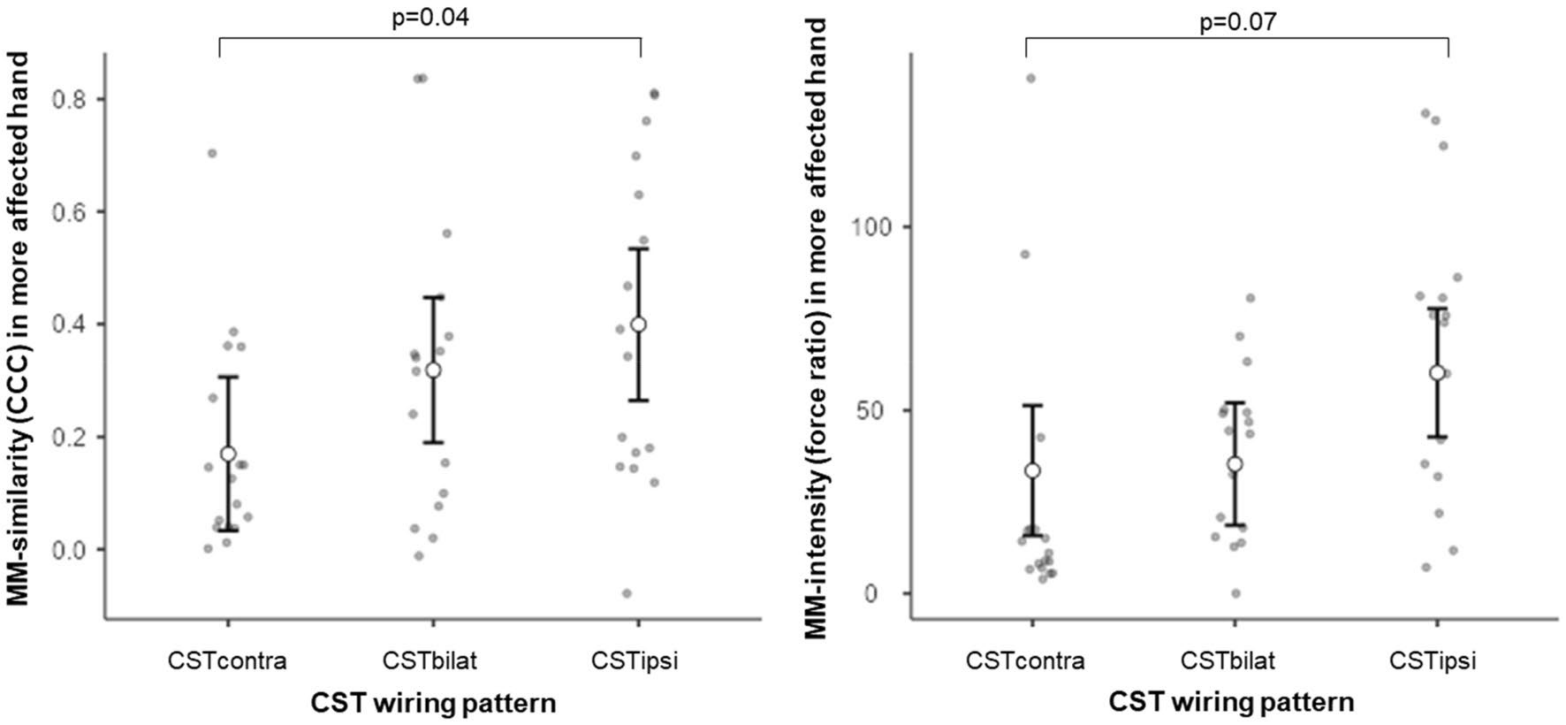
Children with CST ipsi showed the highest MM-similarity (multiple regression analysis, R^2^ = 0.24, p = 0.04, Std. est. = 0.81, p = 0.04) and intensity (multiple regression analysis, R^2^ = 0.30, p = 0.009, Std. est. = 0.68, p = 0.07). Black dot and bar indicate mean and 95% confidence interval, respectively, from the estimated marginal means.

In the more-affected hand, only the CST wiring pattern explained both the high MM-similarity (R^2^ = 0.24, p = 0.04, Std. est. = 0.81, p = 0.04, Fig. 1; Table 4) and high MM-intensity (R^2^ = 0.30, p = 0.009, Std. est. = 0.68, p = 0.07, Fig. 1). Post-hoc analyses showed that children with CSTipsi had higher MM-similarity and intensity compared to the CST contra group.

**Table 4:** Overview of the results obtained in the multiple linear regression analyses for all MM characteristics in each hand.

		R^2^, p-value	Retained predictors (Std. Est, p-value)
**More affected hand**	MM-similarity	R^2^ = 0.24, p = 0.04	CST wiring pattern (CSTipsi-CSTcontra, 0.81, 0.04)
	MM-intensity	R^2^ = 0.30, p = 0.009	CST wiring pattern (CSTipsi-CSTcontra, 0.68, *0.07*)
**Less affected hand**	MM-similarity	R^2^ = 0.67, p < 0.001	CST wiring*Lesion timing (CSTbilat-CSTcontra*CSC-PVL, − 2.18, < 0.001; CSTipsi-CSTcontra*CSC-PVL, − 2.30, < 0.001) CST wiring*Asymmetry index (CSTbilat-CSTcontra*Asymmetry index, − 0.52, 0.06; CSTipsi-CSTcontra*Asymmetry index, − 0.77, 0.03) Lesion timing (CSC-PVL, 1.31, 0.006) CST wiring pattern (CSTbilat-CSTcontra, − 0.96, 0.002; CSTipsi-CSTcontra, 1 + .38, < 0.001) Asymmetry index (0.04, 0.81)
	MM-intensity	R^2^ = 0.33, p < 0.001	Damage to the CC (0.39, 0.007)

All models include manual ability (MACS levels) and age to account for potential confounding factors.

MM, mirror movements; CST, corticospinal tract.

**Figure 2:**
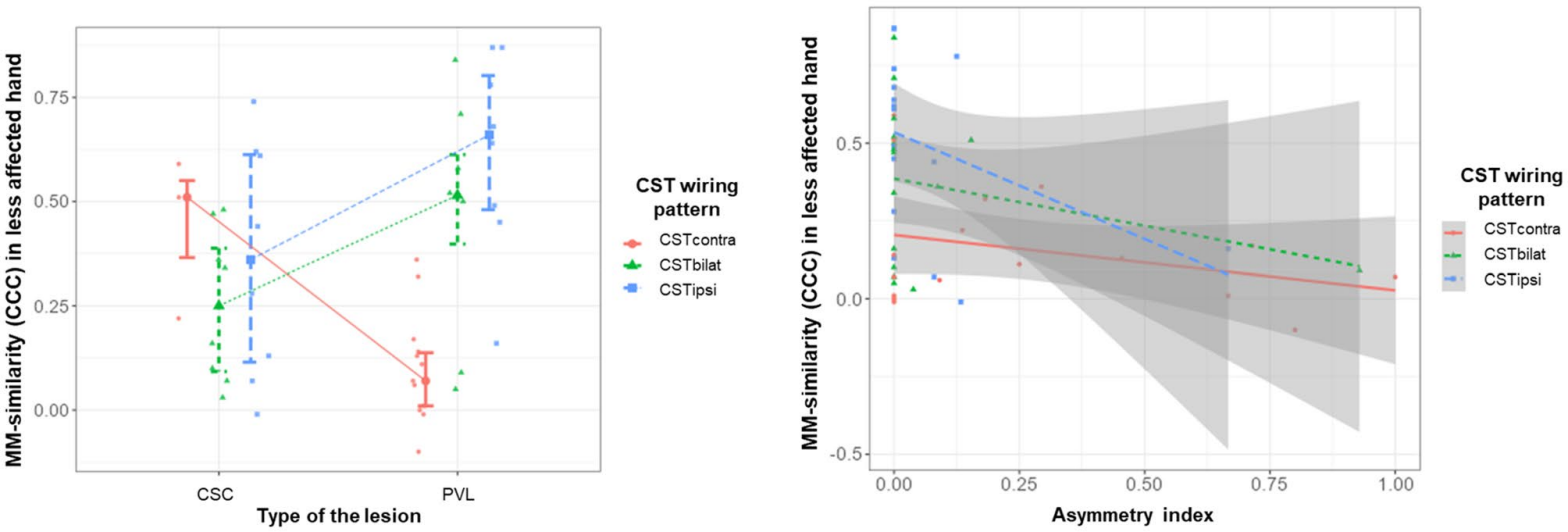
Interactions between neurological factors to explain MM-similarity in the less affected hand. The left panel shows that children with CST contra and CSC lesion have higher MM-similarity compared to those with PVL lesion (multiple regression analysis, Std. est. = − 2.30, p < 0.001), and that children with CST bilat and PVL lesion have higher MM-similarity than those with CSC lesion (multiple regression analysis, Std. est. = − 2.18, p < 0.00). Colored dots and bar indicate mean and 95% confidence interval, respectively, from the estimated marginal means. The right panel shows that children with CST bilat and CST ipsi have higher MM-similarity with more lateralized lesions, compared to children with CST contra (multiple regression analysis, CSTbilat-CSTcontra*Asymmetry index, Std. est. = − 0.52, p = 0.06; CSTipsi-CSTcontra*Asymmetry index, Std. est. = − 0.77, p = 0.03).

**Figure 3:**
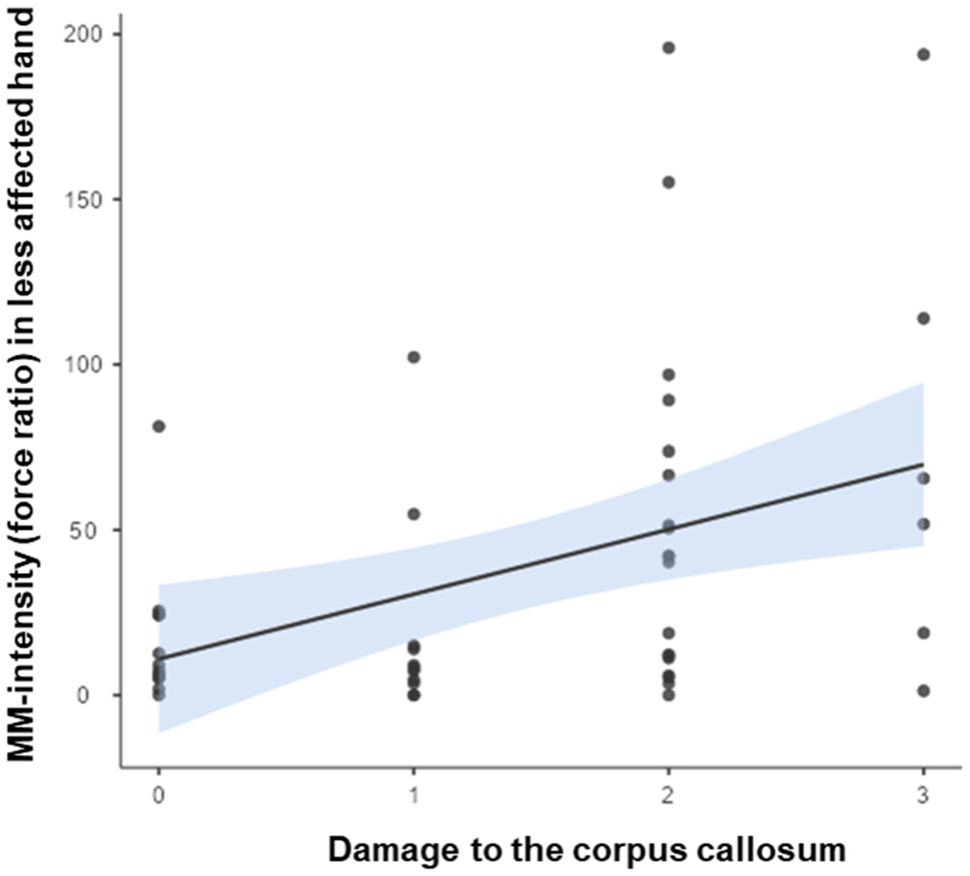
Larger damage to the corpus callosum explained higher MM-intensity in the less-affected hand (multiple regression analysis, R^2^ = 0.33, p < 0.001, Std. est. = 0.39, p = 0.007). Each data point represents a single participant, the line represents the mean and the shaded area is the standard error.

In the less-affected hand, the model was fitted with a larger and more complex number of factors (Table 4). MM-similarity (R^2^ = 0.67, p < 0.001) was explained by (1) an interaction between the CST wiring pattern and the type of the lesion and (2) an interaction between the CST wiring pattern and the asymmetry index. The first interaction showed that children with a CSTcontra and a CSC lesion type had the highest MM-similarity (Std. est. = − 2.30, p < 0.001) and that children with CSTbilat with PVL had higher MM-similarity compared to those with CSTbilat and CSC lesion type (Std. est. = − 2.18, p < 0.001, Fig. 2 left panel). The interaction between CST wiring pattern and asymmetry index indicated that, whilst children with CSTcontra show similar MM-similarity irrespective of the asymmetry of their lesion, children with CSTbilat and CSTipsi show higher MM-similarity when the lesion is more lateralized (index closer to 0; CSTbilat-CSTcontra*Asymmetry index, Std. est. = − 0.52, p = 0.06; CSTipsi-CSTcontra*Asymmetry index, Std. est. = − 0.77, p = 0.03, Fig. 2 right panel). MM-intensity was explained by a single factor, indicating that the larger the damage to the corpus callosum, the higher the intensity (R^2^ = 0.33, p < 0.001, Std. est. = 0.39, p = 0.007, Fig. 3).

## Discussion

In this study, we investigated the relationship between brain lesion characteristics and CST wiring pattern on MM characteristics in each hand in children with uCP. The strength of this study lies in the use of a quantitative assessment of MM that provides different characteristics of the MM phenomenon (similarity and intensity) and the investigation of the combined value of neurological damage in a large sample size (n = 49).

**Figure 4:**
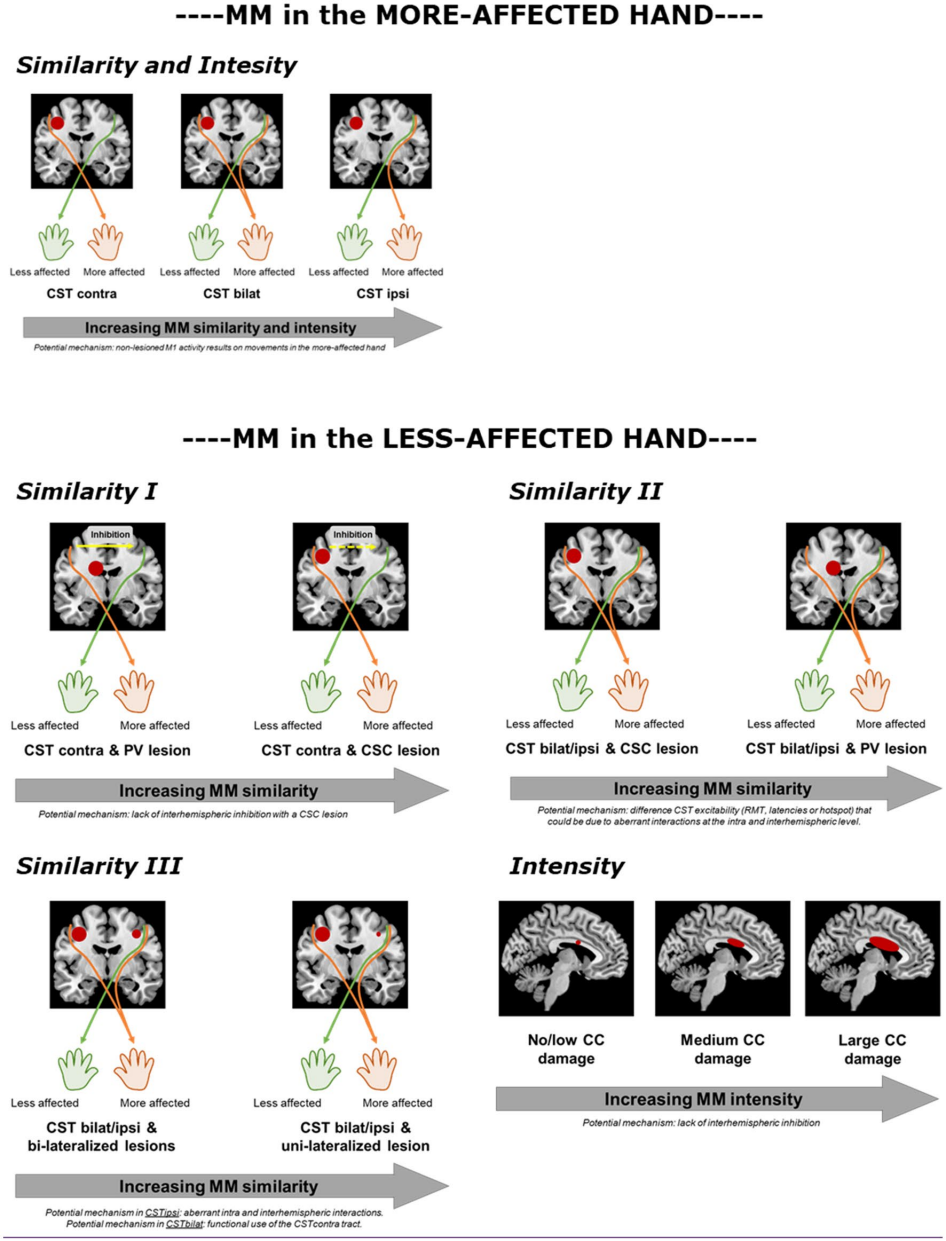
Overview of the results found for each hand and each mirror movement characteristic (similarity and intensity) together with the potential mechanism of such occurrence.

Our results suggest that the underlying neurological mechanism differs according to the hand where the MM occurs, conforming our initial hypothesis. In short, the main underlying factor contributing to MM in the more-affected hand in the CST wiring pattern, whilst MM in the less-affected hand seem to occur due to a more complex interaction of factors including the CST wiring pattern, the lesion timing, as well as items related to lesion location. An overview of the results found for each hand and each MM characteristic (similarity and intensity) together with the potential mechanism of MM occurrence can be found in Fig. 4.

In 1996, Carr et al. hypothesized that an ipsilateral CST projection resulted in stronger MM in the *more-affected hand*^9^. In line, the current study confirmed that more similar and intense MM in the more affected-hand are present in children with a CSTipsi compared to a CSTcontra wiring. While previous literature using the Woods and Teuber scale also supports this finding^12,13,17^, our study provides additional information about MM-similarity and intensity using a quantitative evaluation. MM-similarity has shown to assess the same construct as the Woods and Teuber scale, confirming its validity^18^. Our results, together with the previous literature, suggest that when a child with uCP moves the less-affected hand, the contralesional M1 will activate also the ipsilateral tract that innervates the more-affected hand and, therefore, MM will occur. Whether this activation originates in the same brain area or whether there are intracortical connections that facilitate the mirroring activity is currently unknown.

Interestingly, children with CSTbilat showed more similar MM (although not significant) but not stronger MM compared to CSTcontra (Fig. 1). To innervate their more-affected hand, children with CSTbilat have, besides a contralateral projection, also an ipsilateral one, which may overlap with the motor representation of the contralateral projection from the less-affected hand. The fact that children in this this group may, or may not, have MM could be related to which projection they are using functionally (i.e., the ipsilateral or contralateral projection)^21^, though this requires further investigation.

In the *less-affected hand*, the multifactorial interaction explaining the variability in MM-similarity is more complex. In contrast to our initial hypothesis, MM-similarity was explained by the interactions between (1) CST wiring pattern and lesion type and (2) CST wiring pattern and asymmetry index.

First, children with a CSTcontra had higher MM-similarity in the presence of a CSC compared to a PV lesion. Whilst the presence of an ipsilateral projection (for both CSTbilat and CSTipsi) could explain the presence of MM, as proposed by various studies^12,13,17^, the higher MM-similarity found in the CSTcontra group with a CSC lesion is obviously not due to the presence of an ipsilateral CST. In this case, a lack of interhemispheric inhibition could be the pathophysiological mechanism leading to MM in the less-affected hand^8^. These children could have a simultaneous activation of both primary motor cortices (M1) that could elicit MM due to insufficient interhemispheric inhibition. This mechanism has been shown to be the source of MM in typically developing children^4^ and in adults with congenital MM^22^. Interestingly, lesion location was not retained in the model as a factor related to MM similarity, suggesting that the cortical location of the lesion might not be that relevant. Additionally, children with a CST reorganization, CSTbilat and CSTipsi, and a CSC lesion showed lower MM-similarity compared to children with a PV lesion. In this case, we hypothesize that the functional use of the bilateral or ipsilateral CST tract (i.e., which tract is used to control the movements of the less-affected hand) might influence the similarity of MM, as we found for the more-affected hand. It has previously put forward that latencies of the ipsilateral CST could be an indicator of abnormal CST development^10^, which correlate to poor hand function^23^, and could therefore also relate to MM occurrence^6^. However, these studies focused on the presence of an ipsilateral CST, whilst we also categorized children with a bilateral CST wiring. We hypothesize that these differences could be due to the underlying characteristic of the CST excitability, as it has been shown in patients with bilateral CP^24^: hotspot location activating the functional tract (either contralateral or ipsilateral), resting motor threshold difference on each tract, or longer or shorter latency. Why this differs on the type of the lesion is unknown and, with the current study methodology, we can only formulate a hypothesis on the potential role of aberrant interactions at the interhemispheric or intrahemispheric level, which have been shown to have an influence when there is lateralization of the motor cortex^25^. To unravel these questions, future studies should include neurophysiological techniques of interhemispheric inhibition and intracortical interactions and confirm these hypotheses.

The second interaction showed that MM-similarity of children with CSTcontra were independent of their lesion asymmetry, whilst the MM were more similar with more lateralized lesions in children with CSTipsi and CSTbilat. It is well known that up to 50% of the children with uCP have bilateral lesions despite clearly lateralized impairments^26^ (i.e., hemiparesis). In a TMS study in infants, Eyre et al. found that those with bilateral lesions had a normal pattern of neurophysiological development^27^. Whilst our study results cannot provide with the final answer, we hypothesize that this interaction could be due to the fact that the children with bilateral lesions and CSTbilat wiring could be using the contralateral CST tract functionally, which would result in lower MM occurrence. In children with CSTipsi, it seems plausible that children with more uni-lateralized brain damage may have different cortico-cortical and interhemispheric connectivity compared to those with bilateral damage, which may affect the occurrence of MM. The bilateral damage may trigger both hemispheres to create bypasses and additional connections to thrive beyond the damaged tissue, whilst the unilateral damage may impose the less-affected hemisphere to reorganize, causing MM. Supporting this hypothesis, Eyre et al.^10^ showed a similar pattern of reorganization in children with uCP due to unilateral lesions compared to children with bilateral lesions (potentially leading to bilateral CP).

Lastly, stronger MM were found in the less-affected hand with increasing damage of the corpus callosum. These results are in agreement with our hypothesis and those proposed in previous literature^9^. The corpus callosum is a white matter bundle that acts as the main source of interhemispheric connectivity. Weinstein et al.^28^ showed that stronger MM were related to a decreased integrity of transcallosal fibers, suggesting that a lack of interhemispheric inhibition might be a possible mechanism underlying an increased MM-intensity in children with uCP. Similarly, in children with a bilateral CP, a damaged corpus callosum and a decreased interhemispheric inhibition resulted in more MM^15^. It has been shown that the structural aspects of the corpus callosum, more specifically the callosal motor fibers, are linked to the degree of interhemispheric inhibition^15,29^. Future studies should focus on subparts of the corpus callosum that connect both primary motor cortices or premotor cortices^15^ or other neurophysiological techniques (e.g., interhemispheric inhibition) that can further inform causality^30^.

MM have typically been scored with the Woods and Teuber scale^5^ that evaluates how repetitive the mirroring movement is. We consider this scale to rather evaluate the strength of the mirroring activity, thus the MM-intensity parameter. However, the use of force-based measures allows us to extract other parameters like MM-similarity, that quantify the timing of the mirroring activity. We believe that both parameters are important to evaluate MM as they provide a comprehensive view of the mirroring activity. As such, an activity that shows high similarity (or coupling between hands) with either low or high strength should be considered MM, whilst an activity that shows low similarity (or coupling between hands) with either low or high strength would rather be considered other types of motor overflow^31^.

This study only included children with uCP resulting from PV and CSC lesions, which precludes the generalization of results to children with malformations or postnatally acquired lesions. The inclusion of multiple factors in the analyses also resulted in few children when divided into groups according to the different neurological factors. Therefore, current study results should be replicated in a larger sample. Additionally, more advanced methods to quantify lesion size and the integrity of the corpus callosum may be more appropriate and precise to study the link to MM. In this study, we did not examine the possible role of genetic factors in determining MM in children with uCP^32^. These also require further investigation. The analysis of TMS-derived data (hotspot location, threshold, MEP amplitude, latency) will ensure novel insights in their relation to the MM phenomenon, especially in the groups with CSTipsi and CSTbilateral wiring pattern as this would throw light onto whether a tract is implicated in the occurrence of MM. Future studies of MM in children with uCP should focus on the evaluation of interhemispheric facilitation and inhibition paradigms to investigate to what extent MM occurrence depends on the interhemispheric connectivity, while taking into account the CST wiring pattern and the presence and type of bilateral lesions.

This is the first study to investigate MM in relation to the underlying brain lesion that used a quantitative approach to evaluate MM, allowing identification of both similarity and intensity of the MM. The evaluation of MM with the Windmill task not only gives additional information about the different MM features but has also been shown to additionally increase the sensitivity and specificity of the MM assessment^18^. We encourage future studies to quantitatively evaluate MM providing different features of MM that can deepen our understanding. Current and future investigations in the underlying pathophysiology of MM will assist in the development of new treatment strategies to target the underlying mechanism.

## Conclusion

This study advances our understanding in the mechanisms of MM occurrence in children with uCP. Whilst the CST wiring pattern seems to be the most important factor for MM-similarity and MM-intensity in the more-affected hand, MM characteristics in the less-affected hand seem to be due to a more complex multifactorial problem including intracortical and interhemispheric aspects.

## Methods

### Participants

Children with congenital uCP, aged between 5 and 15 years were recruited from the CP care program of the University Hospitals Leuven (Belgium) and Monash Children’s Hospital (Melbourne, Australia) from June 2015 until November 2017. Children with uCP were included if they were sufficiently mentally capable and cooperative to understand and complete the test procedures. Children were excluded if they (1) received upper limb Botulinum toxin injections six months prior to testing, (2) underwent UL surgery two years prior to testing or (3) had MRI- or TMS-related exclusion criteria (i.e., shunt or any sort of metal in the body, not controlled epilepsy). Whilst we refer to more and less-affected hand, all children had a diagnosis of uCP. The topographical classification of CP (unilateral or bilateral) is given by the clinical signs and symptoms and not by the used brain imaging technique. We opted for this terminology, as there are recent studies showing that the ‘non-affected’ hand (as previously named) is also affected, even slightly^33^. All children verbally assented to participate in the study and all parents signed a written consent form prior to participation in accordance with the Declaration of Helsinki. The study was approved by the local Ethics Committees (Leuven: S55555 and S56513; Monash Health HREC: 12167B).

### Assessments

Children recruited at the University Hospitals Leuven (Belgium) underwent Magnetic Resonance Imaging (MRI) for the purposes of this study. At Monash Children’s Hospital (Australia), the child’s most recent clinical MRI scan was used for this study. All participants underwent Transcranial Magnetic Stimulation (TMS) and MM testing using a quantitative device. We evaluated manual ability with the Manual Ability Classification System (MACS)^34^ and unimanual capacity with the Jebsen-Taylor Hand Function Test^35^. The latter was only available in the Leuven sample (see Results section). The Jebsen-Taylor Hand Function Test evaluates unimanual dexterity by executing tasks to grasp and release different objects, with each hand, as fast as possible. The time taken to execute each task is recorded and summed for all tasks. We calculated a ratio dividing the time taken with the more-affected hand by the time taken with the less-affected hand, whereby a number close to 1 indicated similar performance and > 1 showed the asymmetry in unimanual capacity between hands.

#### Mirror movements

Mirror movements were quantitatively assessed using the same procedure for both sites following a previously published protocol^18^. The device consists of two grip-force transducers connected to a windmill. Children held the transducers between thumb, index, and middle finger, although they could use more fingers if they had difficulties applying this pinch-grip with their more-affected hand (the grip of the less-affected hand was always matched to the grip of the more-affected hand). First, we measured the maximum voluntary contraction (MVC) of each hand three times and used the average of the three repetitions for analysis. The less-affected hand was always measured first. Second, the children performed the task by repetitively squeezing one of the transducers with one hand, while the other hand was instructed to simply hold onto the other transducer. The transducer that was squeezed actively was connected to a miniature windmill. Once the transducer was pressed beyond a threshold of at least 15% of the MVC and if the grip-force exceeded 1.5 kg, the windmill started rotating. Children were asked to rotate the windmill, which occurred when executing a repetitive squeezing pattern with a frequency of ≥ 1 Hz. A trial consisted of a squeezing period of 5 s. The force profiles (mV/V) during 20 trails (10/hand) were collected using PsychoPy standalone version 1.82^19^ and further used to extract the MM characteristics (see Data analysis).

#### Magnetic resonance imaging

Magnetic resonance imaging was collected in Leuven (Belgium) with a 3 T Philips Ingenia scanner (Philips Healthcare, Best, the Netherlands) with a protocol detailed elsewhere^36^. Briefly, we acquired a T1-weighted 3D fluid attenuated inversion recovery (3D FLAIR: 321 sagittal slices, slice thickness = 1.2 mm, slice gap = 0.6 mm, repetition time = 4800 ms, echo time = 353 ms, field of view = 250 × 250 mm^2^, 1.1 × 1.1 ×   0.56 mm^3^ voxel size, acquisition time = 5 min) image and a magnetization prepared rapid gradient echo (MPRAGE: 182 slices, slice thickness = 1.2 mm, slice gap = 0 mm, TR = 9.7 ms, TE = 4.6 ms, FOV: 250 × 250 mm^2^, 0.98 × 0.98 × 1.2 voxel size, acquisition time = 6 min)^36^. Prior to data acquisition, children were familiarized with the scanner by performing scan-related tasks (e.g., laying still, wearing a helmet and earplugs). At Monash Children’s Hospital (Australia), existing 3DFLAIR and/or MPRAGE MRI images were retrieved from the medical records if they were collected after the age of 3 years, as the myelination process is then complete^37^.

Brain lesion characteristics (lesion location and extent) were identified by an experienced child neurologist (EO). Brain lesion type was classified into three groups: brain malformations, PV and CSC lesions^38^. The quantification of the brain lesion characteristics was performed with a valid and reliable semi-quantitative scale^39,40^ containing a graphical template of the lobes, corpus callosum cerebellum and subcortical structures. First, the MRI slices that corresponded to the template were identified and the lesion was drawn by a child neurologist experienced in the use of the scale (EO).

First, we extracted information on *lesion location*, that was indicated by the damage to the frontal and parietal lobes (scores 0–4), the basal ganglia and thalamus (scores 0–3) and the corpus callosum (scores 0–3, according to the number of thirds that were damaged). Also, we documented the presence of bilateral lesions or purely unilateral lesions and calculated an asymmetry index as the ratio between the total contralesional (less-affected hemisphere) damage divided by the total ipsilesional (more-affected hemisphere) damage. An index of 0 indicated a purely unilateral lesion, whilst an index of 1 indicated that both hemispheres were equally affected. Regarding *lesion extent*, we calculated the score of the total ipsilesional damage (scores 0–17) based on the damage to all lobes (scores 0–3 for each lobe, i.e., total of 0–12) and the subcortical structures (scores 0–5 including the lenticular, caudate, posterior limb of the internal capsule (PLIC), thalamus and brainstem).

#### Transcranial magnetic stimulation

TMS was used to identify the underlying CST wiring pattern with a previously described protocol^36^. We used a Magstim 200 Stimulator (The Magstim Company Ltd, Whitland, Wales, UK) with a focal 2 × 70 mm figure-eight coil to evoke motor evoked potentials (MEPs) on the adductor pollicis brevis muscle in both hands. The child sat on a chair with both hands relaxed on a cushion. A cap was placed on the head on which a raster was drawn to help with the identification of the optimal location for evoking an MEP (≥ 50 µV amplitude) for each hand (i.e., hotspot). We started with an intensity of 30% which was gradually increased until a MEP was evoked (resting motor threshold, RMT). Next, 10 MEPs were collected at 120% of the RMT. If the RMT was 85% or higher, 10 MEPs were collected at maximum stimulator output (100%). We first identified the CST from the less-affected hemisphere to the less-affected hand, followed by the ipsilateral CST (innervating the more-affected hand), which was identified and measured separately. Lastly, we stimulated the more-affected hemisphere to search for a remaining contralateral CST. This protocol indicated the CST wiring pattern, i.e., contralateral (CSTcontra, the more-affected hand receives input from the crossed CST, originating in the more-affected hemisphere), ipsilateral (CSTipsi, the more-affected hand receives input from the uncrossed CST, originating in the less-affected hemisphere), or bilateral (CSTbilat, the more-affected hand receives input from both the crossed and uncrossed CSTs, originating in the more-affected and less-affected hemispheres, respectively). In this study, the TMS evaluation was used for diagnostic purposes. When high intensities were not tolerated, we increased the intensity up to at least 80% of the maximum stimulator output and the participants were asked to hold a pen to precontract the evaluated muscle and facilitate the CST and MEP detection. This allowed us to rule out the possibility of mis-categorizing the participant regarding their CST wiring pattern^36^. There were no adverse events.

### Data analysis

#### Mirror movements

A custom-written script was used to analyse the MM data using a Psychopy standalone version 1.82 software. First, we visually inspected the 20 trials per child (10 trials per hand). One trial displayed graphs of the grip forces of both hands. Visual inspection of the trials was needed to identify the valid trials. A valid trial consisted of at least 10 squeezes of the active hand above a threshold of 1.5 kg and with a grip-force of 15% of the child’s MVC. The first 500 ms after the ‘start’ signal were not considered to account for a delay in reaction time. Only valid trials were retained for the statistical analysis.

To quantify the MM, both the similarity (i.e. coupling of force profiles) and intensity (i.e. strength of the mirroring activity) were calculated, following the procedures described elsewhere^18,19^. MM-similarity consisted of the maximum correlation-coefficient representing an index of similarity between the squeezing signals of both hands (range from 0–1, ‘0’ indicating no MM and ‘1’ mirroring activity identical to the voluntary movements), which was averaged across trials. Second, we calculated the MM-intensity by computing the mean grip force as the difference between the heights and troughs of the force signal and this was averaged across trials. The ratio between the passive and active hand was computed as follows:

$$MM{\text{ - }}intensity = \frac{{{\text{PeakesPassive}} \times {\text{MVCactive}}}}{{{\text{PeakesActive}} \times {\text{MVCpassive}}}} \times 100.$$

A higher MM-intensity ratio indicates more severe MM.

#### Statistical analyses

We checked the data distribution with the Shapiro–Wilk test and visual inspection of the histograms. The MM and unimanual capacity data were not-normally distributed and we used non-parametric statistics to report the comparison and correlations analyses. For the regression analyses, we used parametric statistics and checked that the model residuals had a symmetric distribution.

First, we documented differences in MM data according to more-affected side and sex, and its relationship with age, hand function (MACS levels and Jebsen-Taylor Hand Function test), using non-parametric statistics. Data of the Jebsen-Taylor Hand Function test was only available for the Leuven sample (n = 44). Effect sizes were reported with rank biserial correlation (r_rb_), point biserial correlation (r_pb_) or Spearman’s rho (rho), according to the type of data, and were interpreted as little or no correlation (< 0.30), low (0.30–0.50), moderate (0.50–0.70), high (0.70–0.90), and very high correlation (> 0.90)^41^. Statistical significance for these tests was set at α < 0.05, with pairwise comparisons when applicable (MACS levels) using the Tukey test. Second, descriptive statistics were used to document the distribution of MM characteristics according to the brain lesion characteristics and the CST wiring pattern. Third, we investigated the association between *each* brain lesion characteristic and the CST wiring pattern on MM characteristics in each hand using simple linear regression, controlling for manual ability (MACS levels) and age. Next, we explored to what extent MM characteristics in each hand could be explained by the brain lesion characteristics, and their interaction with the CST wiring pattern in a multiple regression analysis. This analysis was fitted with the backward elimination method until we identified a set of variables significantly contributing to the model, and R^2^ (that also served as effect size) and we report standardized beta coefficients and p-values. The standardized beta coefficients describe the strength of the effect of the specific neurological factor on the MM characteristics. The larger its absolute value, the larger the effect, and their size is comparable between factors. Those factors that did not influence (p < 0.05) any MM characteristic in any hand in the simple linear regression analyses were not included in the multiple linear regression model. Statistical significance was set at α < 0.01, to correct for the high number of statistical tests performed in this study.

All statistical analyses were computed with SPSS Statistics for Windows version 25.0 (IBM Corp., Armonk, NY) and jamovi for Windows version 1.6.3^42^.

## Supplementary Information

Supplementary Information.

## Data Availability

The data that support the findings of this study are available on request from the corresponding author, [CSM].

## References

[1] Adler C, Berweck S, Lidzba K, Becher T, Staudt M (2015). Mirror movements in unilateral spastic cerebral palsy: Specific negative impact on bimanual activities of daily living. Eur. J. Paediatr. Neurol..

[2] Koerte I (2010). Mirror movements in healthy humans across the lifespan: Effects of development and ageing. Dev. Med. Child Neurol..

[3] Kuhtz-Buschbeck JP, Sundholm LK, Eliasson AC, Forssberg H (2000). Quantitative assessment of mirror movements in children and adolescents with hemiplegic cerebral palsy. Dev. Med. Child Neurol..

[4] Mayston MJ, Harrison LM, Stephens JA (1999). A neurophysiological study of mirror movements in adults and children. Ann. Neurol..

[5] Woods BT, Teuber HL (1978). Mirror movements after childhood hemiparesis. Neurology.

[6] Klingels K (2016). Do mirror movements relate to hand function and timing of the brain lesion in children with unilateral cerebral palsy?. Dev. Med. Child Neurol..

[7] Simon-Martinez C (2020). Randomized controlled trial combining constraint-induced movement therapy and action-observation training in unilateral cerebral palsy: Clinical effects and influencing factors of treatment response. Ther. Adv. Neurol. Disord..

[8] Kuo HC, Friel KM, Gordon AM (2018). Neurophysiological mechanisms and functional impact of mirror movements in children with unilateral spastic cerebral palsy. Dev. Med. Child Neurol..

[9] Carr LJ (1996). Development and reorganization of descending motor pathways in children with hemiplegic cerebral palsy. Acta Paediatr..

[10] Eyre JA (2007). Corticospinal tract development and its plasticity after perinatal injury. Neurosci. Biobehav. Rev..

[11] Holmström L (2010). Hand function in relation to brain lesions and corticomotor-projection pattern in children with unilateral cerebral palsy. Dev. Med. Child Neurol..

[12] Riddell M, Kuo H-C, Zewdie E, Kirton A (2019). Mirror movements in children with unilateral cerebral palsy due to perinatal stroke: Clinical correlates of plasticity reorganization. Dev. Med. Child Neurol..

[13] Rich TL (2020). Ipsilateral corticospinal tract excitability contributes to the severity of mirror movements in unilateral cerebral palsy: A case series. Clin. EEG Neurosci..

[14] Staudt M (2004). Reorganization in congenital hemiparesis acquired at different gestational ages. Ann. Neurol..

[15] Koerte I (2011). Anisotropy of transcallosal motor fibres indicates functional impairment in children with periventricular leukomalacia. Dev. Med. Child Neurol..

[16] Krägeloh-Mann I, Horber V (2007). The role of magnetic resonance imaging in elucidating the pathogenesis of cerebral palsy: A systematic review. Dev. Med. Child Neurol..

[17] Staudt M (2002). Two types of ipsilateral reorganization in congenital hemiparesis: A TMS and fMRI study. Brain J. Neurol..

[18] Zielinski IM (2018). Windmill-task as a new quantitative and objective assessment for mirror movements in unilateral cerebral palsy: A pilot study. Arch. Phys. Med. Rehabil..

[19] Zielinski IM (2017). The relation between mirror movements and non-use of the affected hand in children with unilateral cerebral palsy. Dev. Med. Child Neurol..

[20] Mailleux L (2017). How does the interaction of presumed timing, location and extent of the underlying brain lesion relate to upper limb function in children with unilateral cerebral palsy?. Eur. J. Paediatr. Neurol. EJPN Off. J. Eur. Paediatr. Neurol. Soc..

[21] Simon-Martinez C (2019). Influence of the corticospinal tract wiring pattern on sensorimotor functional connectivity and clinical correlates of upper limb function in unilateral cerebral palsy. Sci. Rep..

[22] Fasano A (2014). Congenital mirror movements in a new Italian family. Mov. Disord. Clin. Pract..

[23] Vandermeeren Y, Bastings E, Fadiga L, Olivier E (2003). Long-latency motor evoked potentials in congenital hemiplegia. Clin. Neurophysiol..

[24] Maegaki Y (1999). Central motor reorganization in cerebral palsy patients with bilateral cerebral lesions. Pediatr. Res..

[25] Pilato F (2020). Multimodal assessment of motor pathways and intracortical connections in functional hemispherectomy. Childs Nerv. Syst..

[26] Holmefur M (2013). Neuroradiology can predict the development of hand function in children with unilateral cerebral palsy. Neurorehabil. Neural Repair.

[27] Eyre JA (2007). Is hemiplegic cerebral palsy equivalent to amblyopia of the corticospinal system?. Ann. Neurol..

[28] Weinstein M (2014). Interhemispheric and intrahemispheric connectivity and manual skills in children with unilateral cerebral palsy. Brain Struct. Funct..

[29] Wahl M (2007). Human motor corpus callosum: Topography, somatotopy, and link between microstructure and function. J. Neurosci..

[30] Eng D, Zewdie E, Ciechanski P, Damji O, Kirton A (2018). Interhemispheric motor interactions in hemiparetic children with perinatal stroke: Clinical correlates and effects of neuromodulation therapy. Clin. Neurophysiol..

[31] Hoy KE, Fitzgerald PB, Bradshaw JL, Armatas CA, Georgiou-Karistianis N (2004). Investigating the cortical origins of motor overflow. Brain Res. Rev..

[32] Franz, E. A. Characterizing the phenotypes of congenital mirror movements and other rare genetic disorders. 10.1111/dmcn.14509. 10.1111/dmcn.14509 32157690

[33] Rich TL, Menk JS, Rudser KD, Feyma T, Gillick BT (2017). Less-affected hand function in children with hemiparetic unilateral cerebral palsy: A comparison study with typically developing peers. Neurorehabil. Neural Repair.

[34] Eliasson ACS (2006). The Manual Ability Classification System (MACS ) for children. Dev. Med. Child Neurol..

[35] Araneda R (2019). Reliability and responsiveness of the Jebsen-Taylor test of hand function and the box and block test for children with cerebral palsy. Dev. Med. Child Neurol..

[36] Simon-Martinez C (2018). Corticospinal tract wiring and brain lesion characteristics in unilateral cerebral palsy: Determinants of upper limb motor and sensory function. Neural Plast..

[37] Welker KM, Patton A (2012). Assessment of normal myelination with magnetic resonance imaging. Semin. Neurol..

[38] Himmelmann K (2017). MRI classification system (MRICS) for children with cerebral palsy: Development, reliability, and recommendations. Dev. Med. Child Neurol..

[39] Fiori S (2014). Reliability of a novel, semi-quantitative scale for classification of structural brain magnetic resonance imaging in children with cerebral palsy. Dev. Med. Child Neurol..

[40] Fiori S (2015). Validity of semi-quantitative scale for brain MRI in unilateral cerebral palsy due to periventricular white matter lesions: Relationship with hand sensorimotor function and structural connectivity. NeuroImage Clin..

[41] Hinkle, D. E. & Wiersma, W. *Applied Statistics for the Behavioral Sciences*. 756 (Houghton Mifflin, 1998).

[42] The jamovi project 2020. (2020).

