# Supplementary material for: The impact of brain lesion characteristics and the corticospinal tract wiring on mirror movements in unilateral cerebral palsy
Source: Sci Rep. 2022 Sep 29;12:16301. doi: 10.1038/s41598-022-19920-z (PMC9522771; doi:10.1038/s41598-022-19920-z)
Supplement: Supplementary file 1 — Supplementary Information. [file 41598_2022_19920_MOESM1_ESM.docx]

**Supplementary Materials**

**Table S1.** Number of children excluded for this study and reasons.

| **Reason for exclusion** | **Number of children** |
| --- | --- |
| MM related |  |
| Poor collaboration with MM evaluation | 2 |
| TMS related |  |
| Refusal to TMS or unable to come back for TMS evaluation | 11 |
| Resting Motor Threshold higher than tolerable | 4 |
| Unable to relax hands during TMS | 2 |
| Unable to elicit Motor Evoked Potentials | 1 |
| VP shunt (contraindication) | 2 |
| MRI/Brain related |  |
| Tumor | 1 |
| No visible lesion in MRI | 2 |
| MRI unavailable | 1 |
| Hemispherectomy | 2 |
| Malformation | 1 |
| Acquired lesion | 4 |
| Mixed reasons |  |
| Malformation and epilepsy (contraindication for TMS) | 1 |
| Malformation and unable to relax hands | 1 |
| Panic attack during MRI and refusal to TMS evaluation | 2 |
| **Total** | **37** |

MRI, Magnetic Resonance Imaging; MM, Mirror Movements; TMS, Transcranial Magnetic Stimulation; VP, ventricular-peritoneal.

**
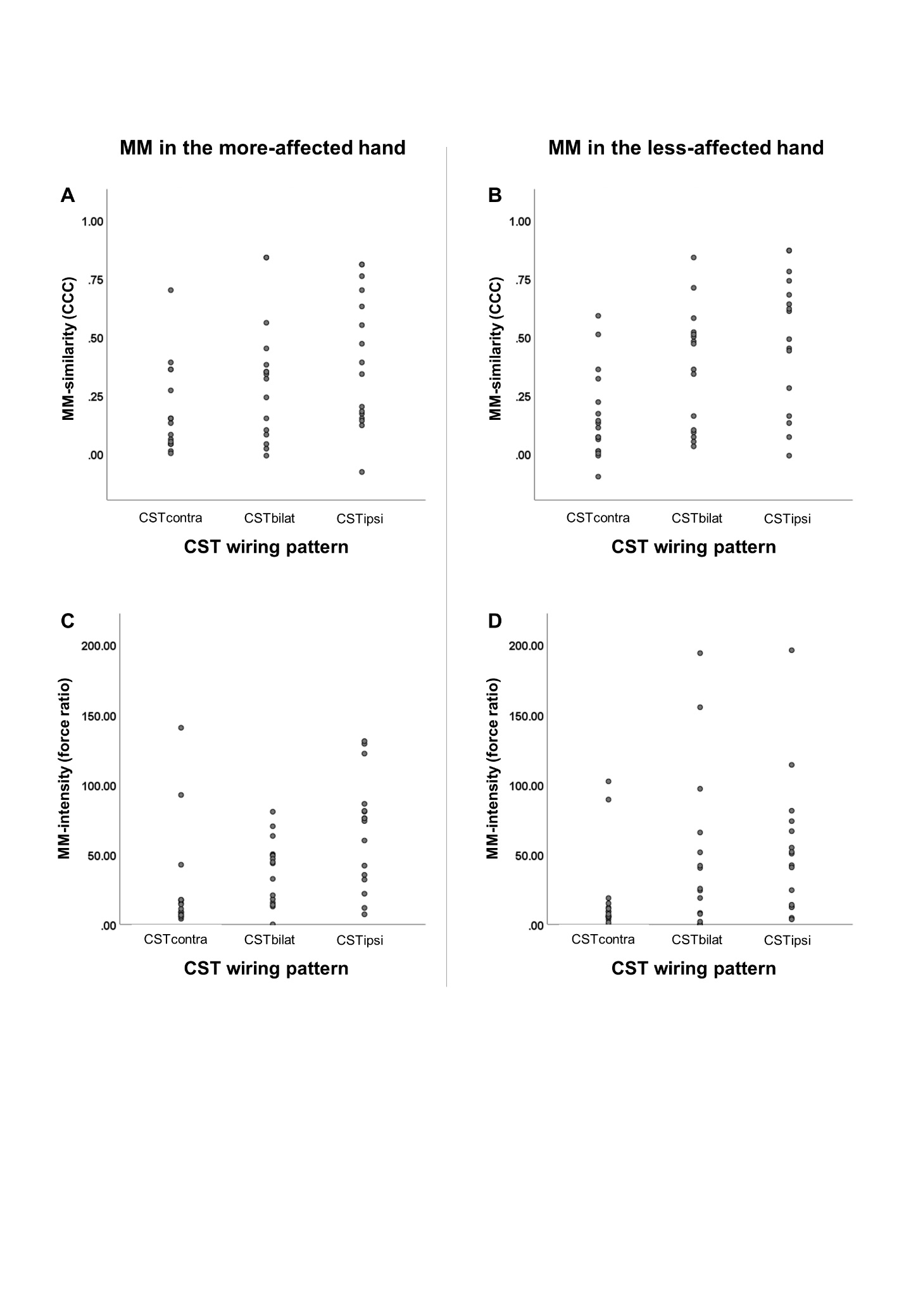
**

**Figure S1**. Individual data points of MM-similarity (A and B) and MM-intensity (C and D) for both the more-affected (left panel) and the less-affected hand (right panel) in relation with the type of corticospinal tract wiring pattern.

**
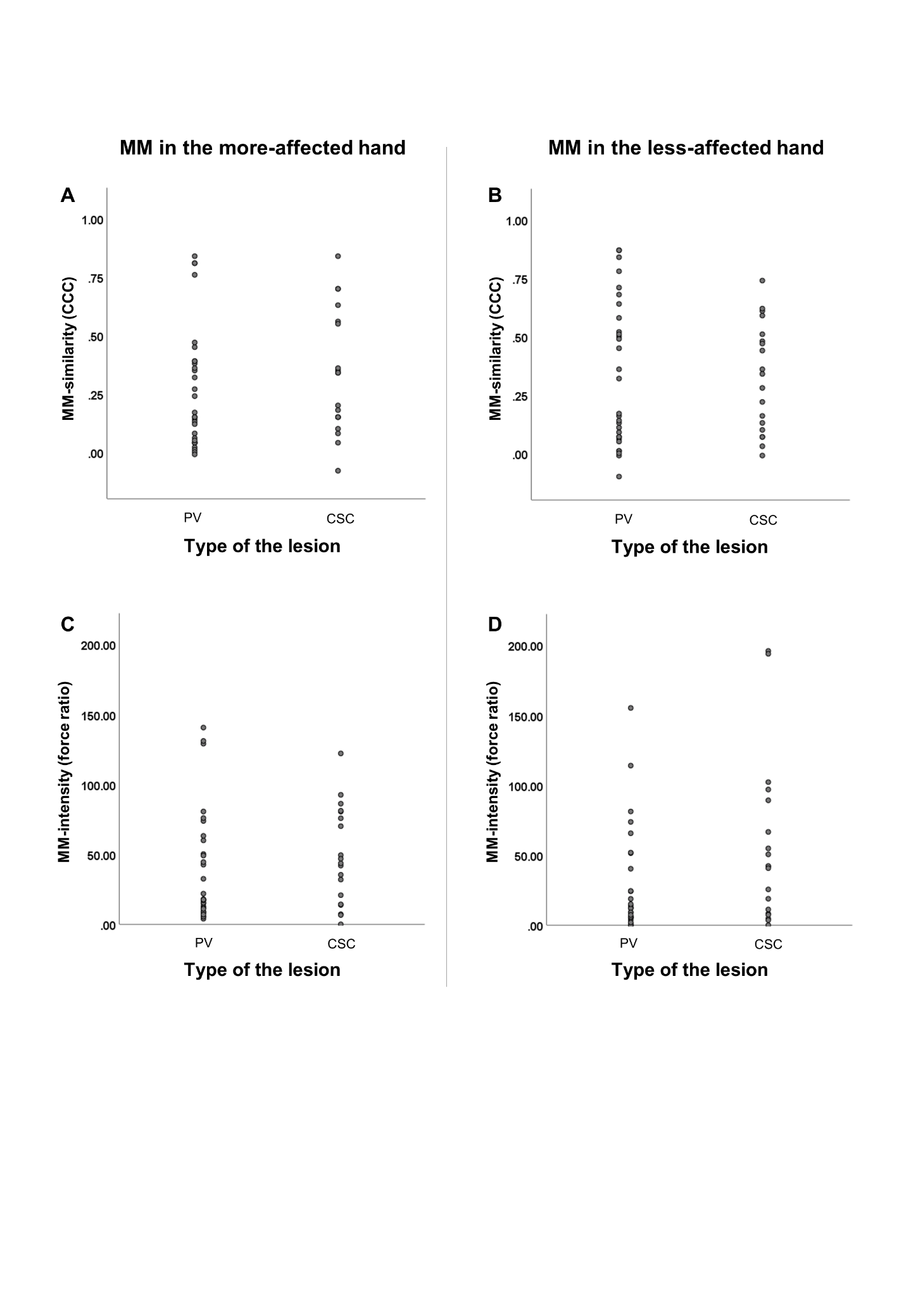
**

**Figure S2**. Individual data points of MM-similarity (A and B) and MM-intensity (C and D) for both the more-affected (left panel) and the less-affected hand (right panel) in relation with the type of the lesion.

**
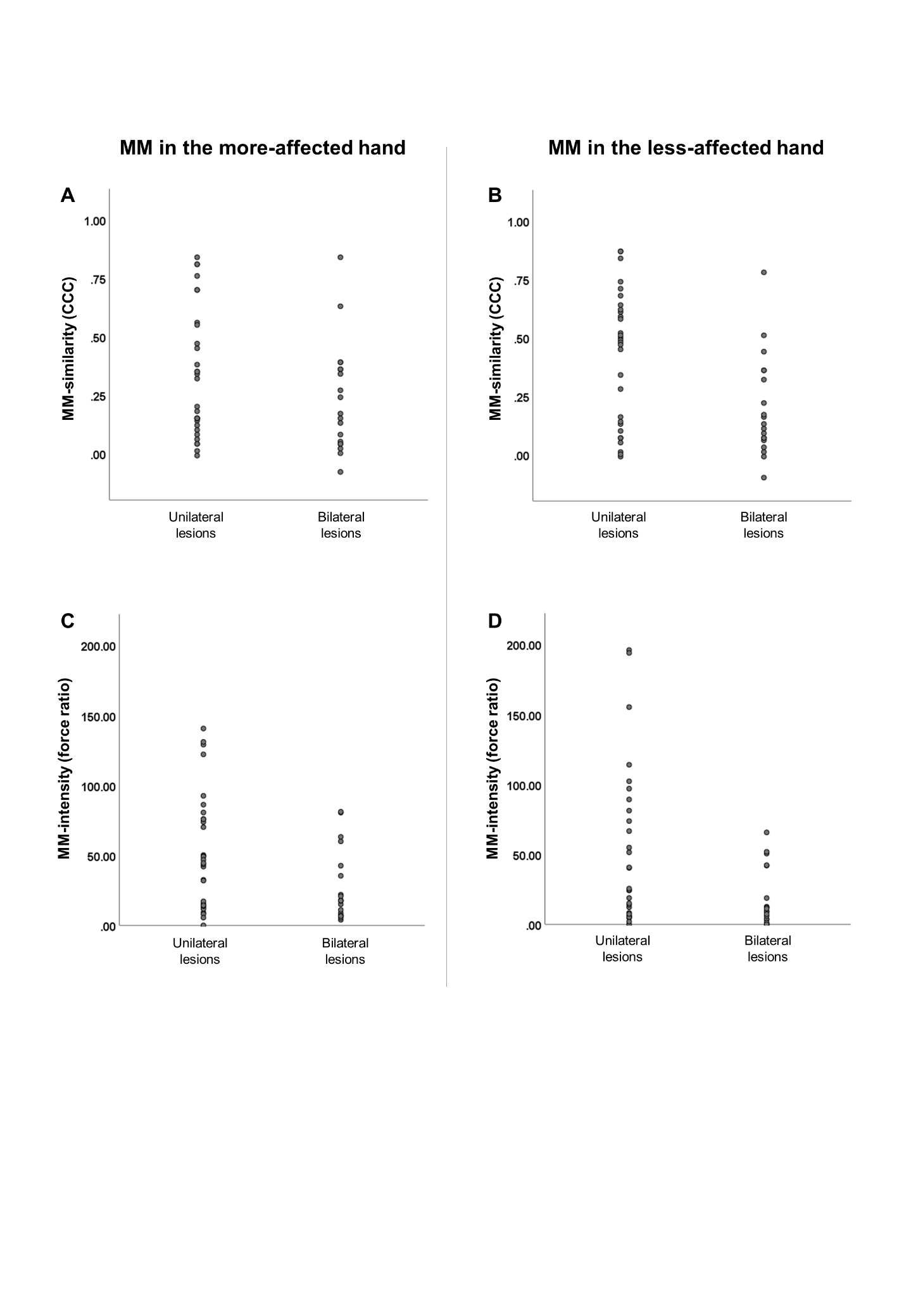
**

**Figure S3**. Individual data points of MM-similarity (A and B) and MM-intensity (C and D) for both the more-affected (left panel) and the less-affected hand (right panel) in relation with the presence of unilateral or bilateral lesions.

**
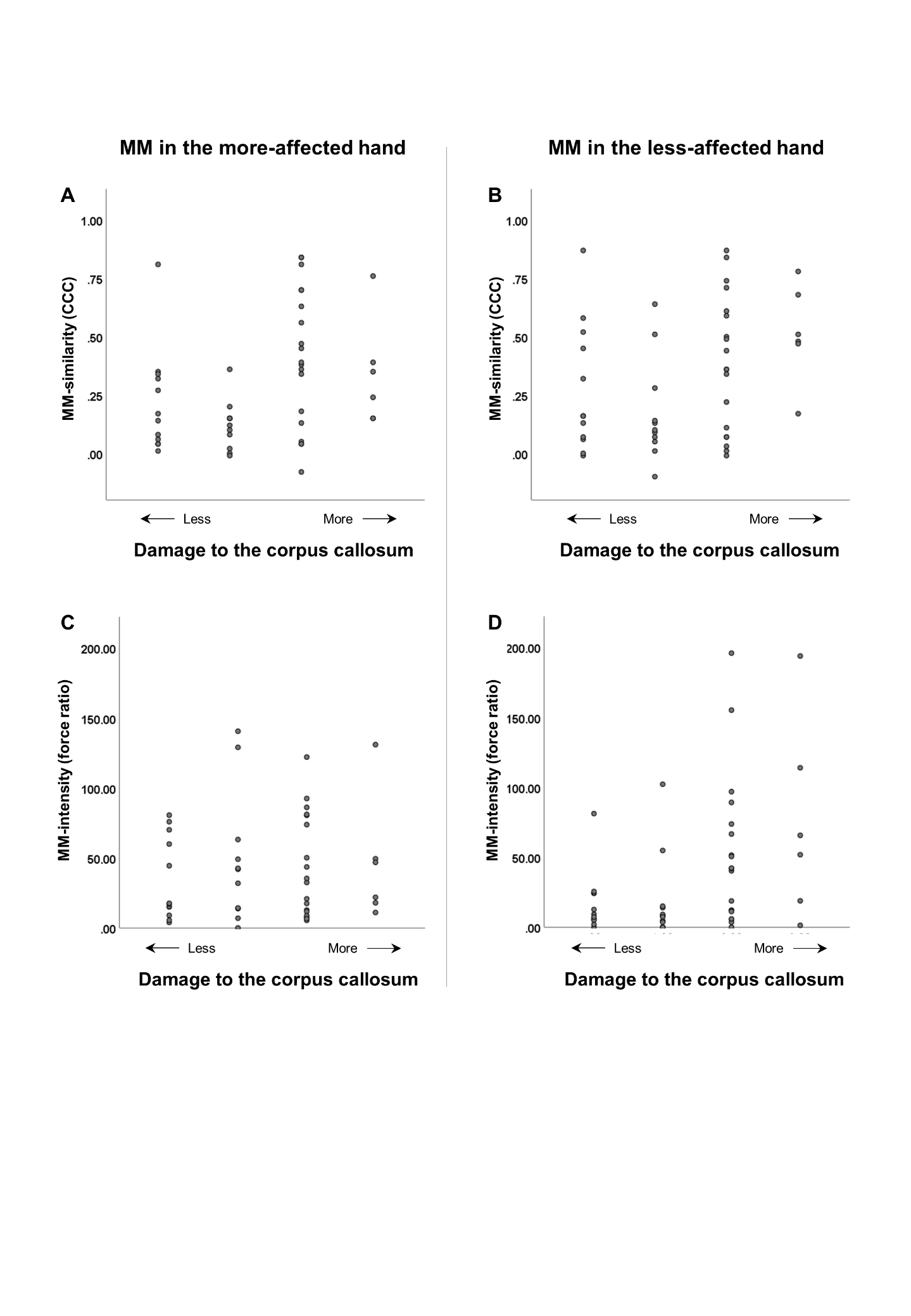
**

**Figure S4**. Individual data points of MM-similarity (A and B) and MM-intensity (C and D) for both the more-affected (left panel) and the less-affected hand (right panel) in relation with the damage to the corpus callosum.

**
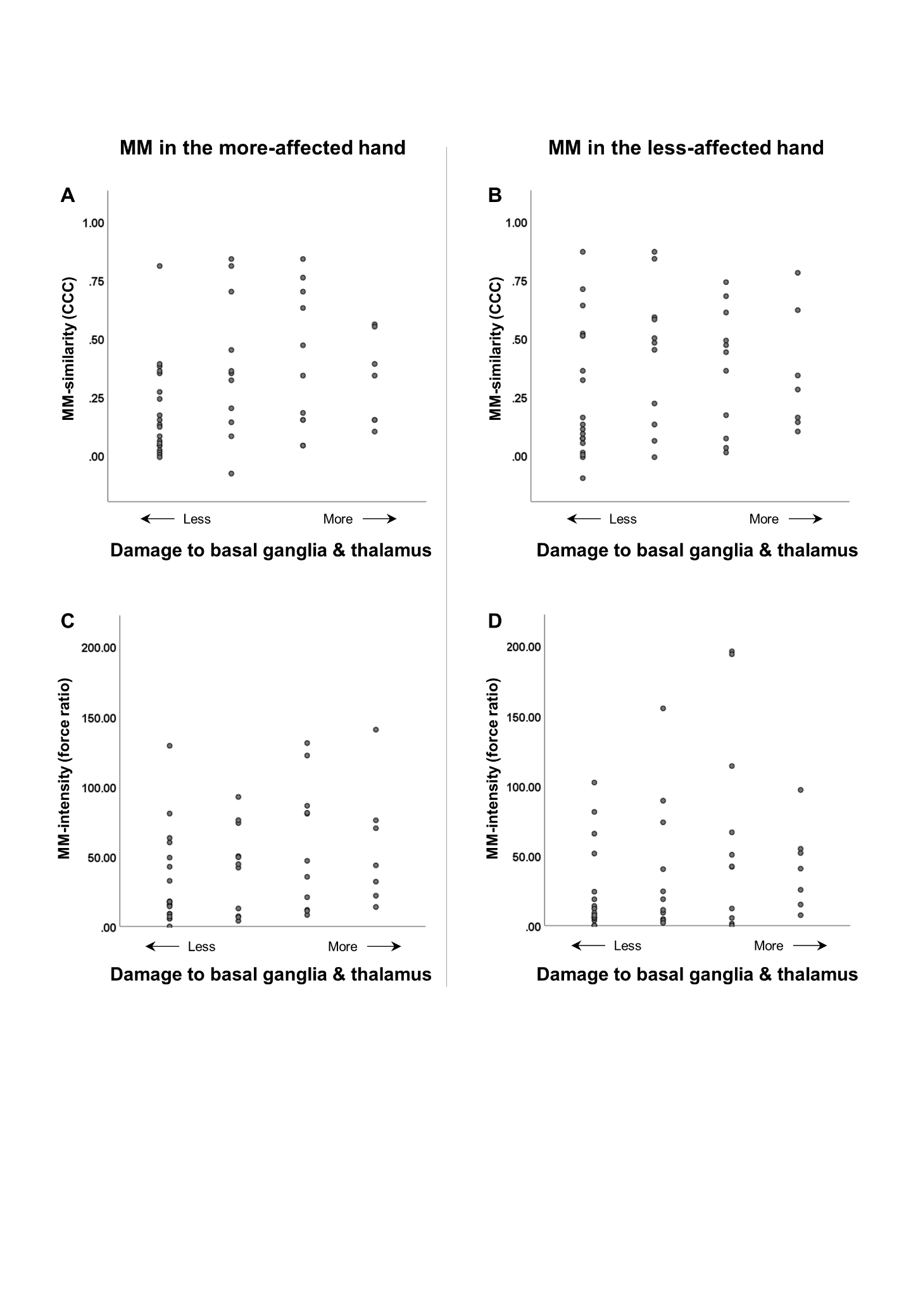
**

**Figure S5**. Individual data points of MM-similarity (A and B) and MM-intensity (C and D) for both the more-affected (left panel) and the less-affected hand (right panel) in relation with the damage to the basal ganglia and thalamus.

**
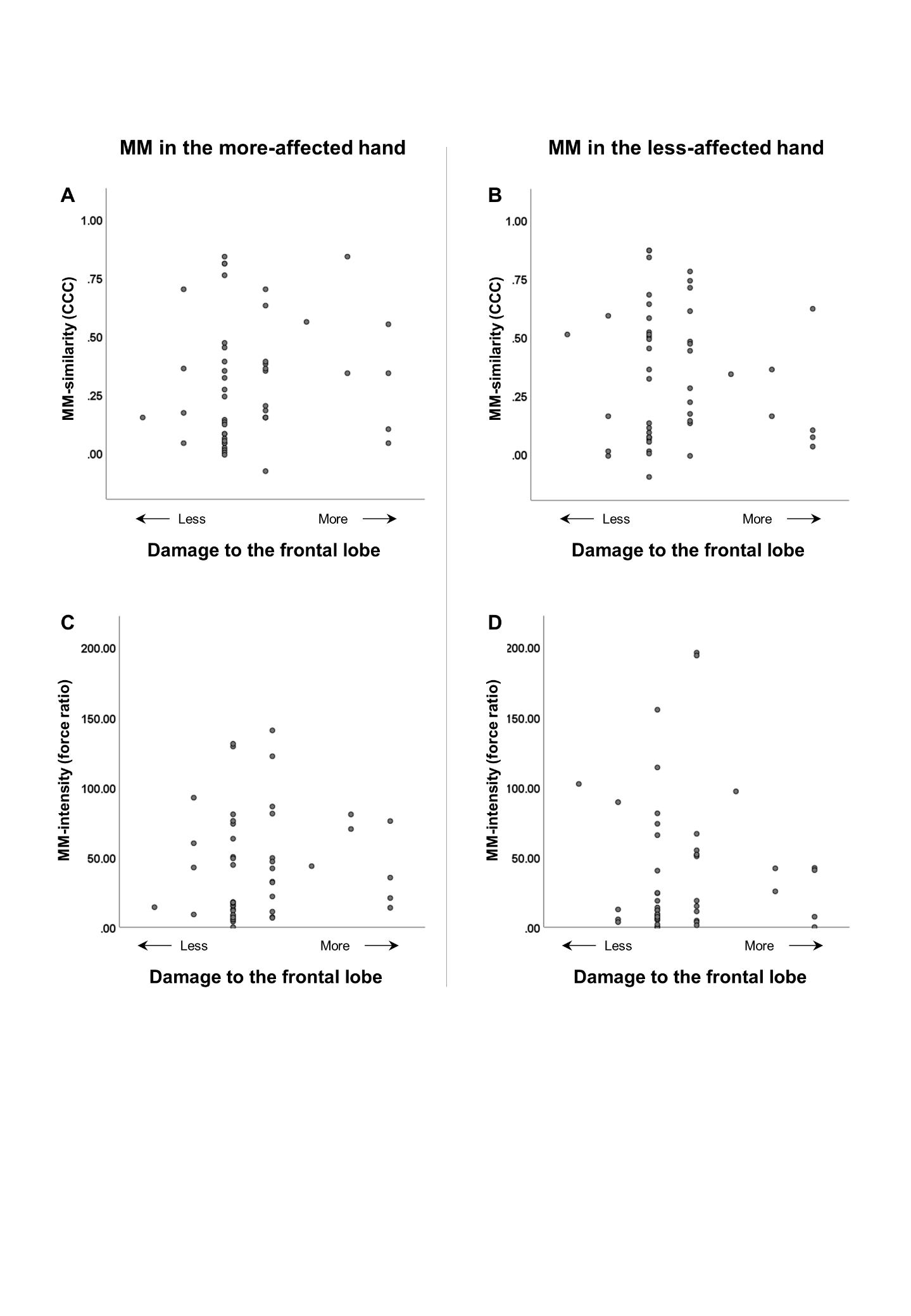
**

**Figure S6**. Individual data points of MM-similarity (A and B) and MM-intensity (C and D) for both the more-affected (left panel) and the less-affected hand (right panel) in relation with the damage to the frontal lobe.

**
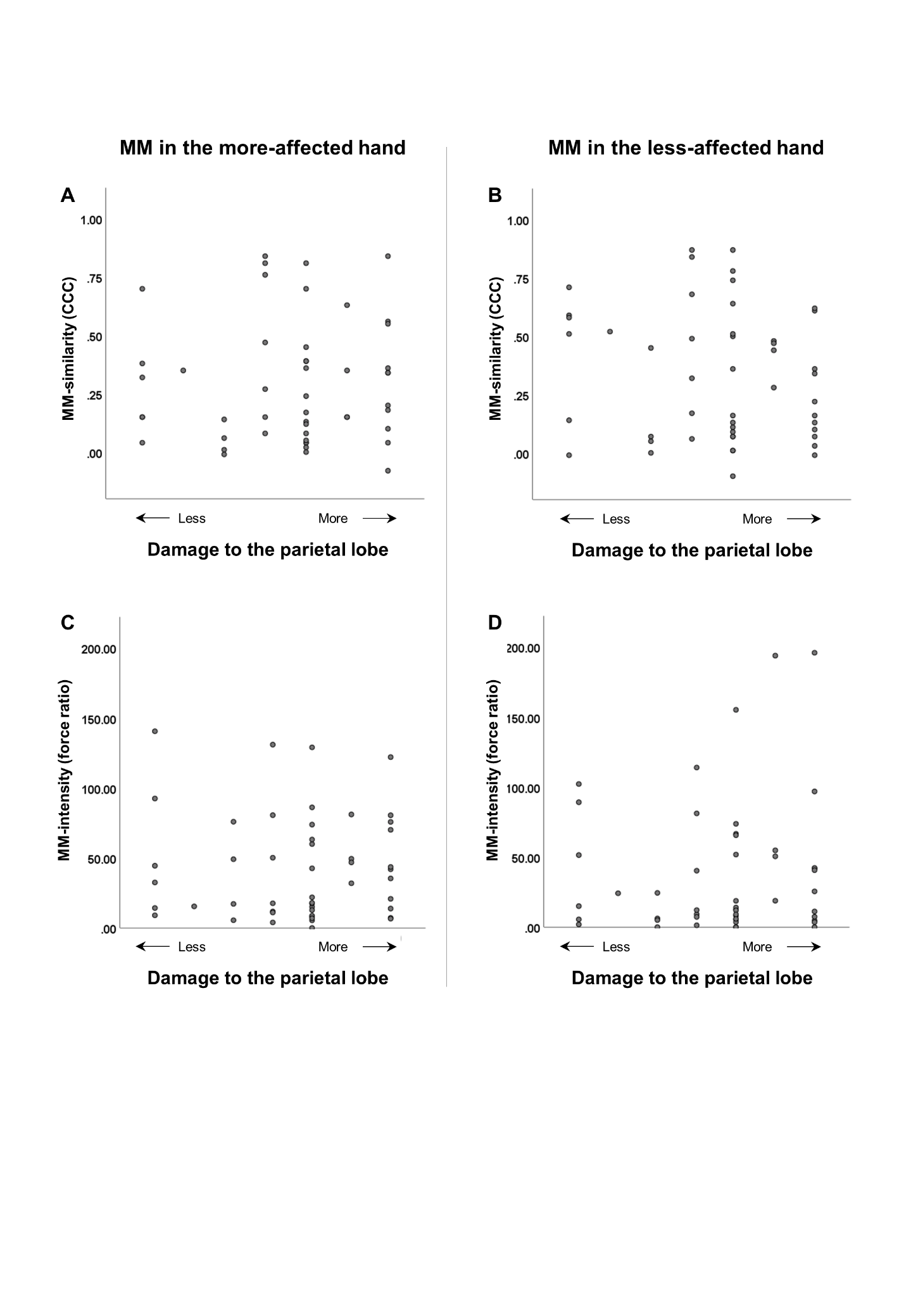
**

**Figure S7**. Individual data points of MM-similarity (A and B) and MM-intensity (C and D) for both the more-affected (left panel) and the less-affected hand (right panel) in relation with the damage to the parietal lobe.

**
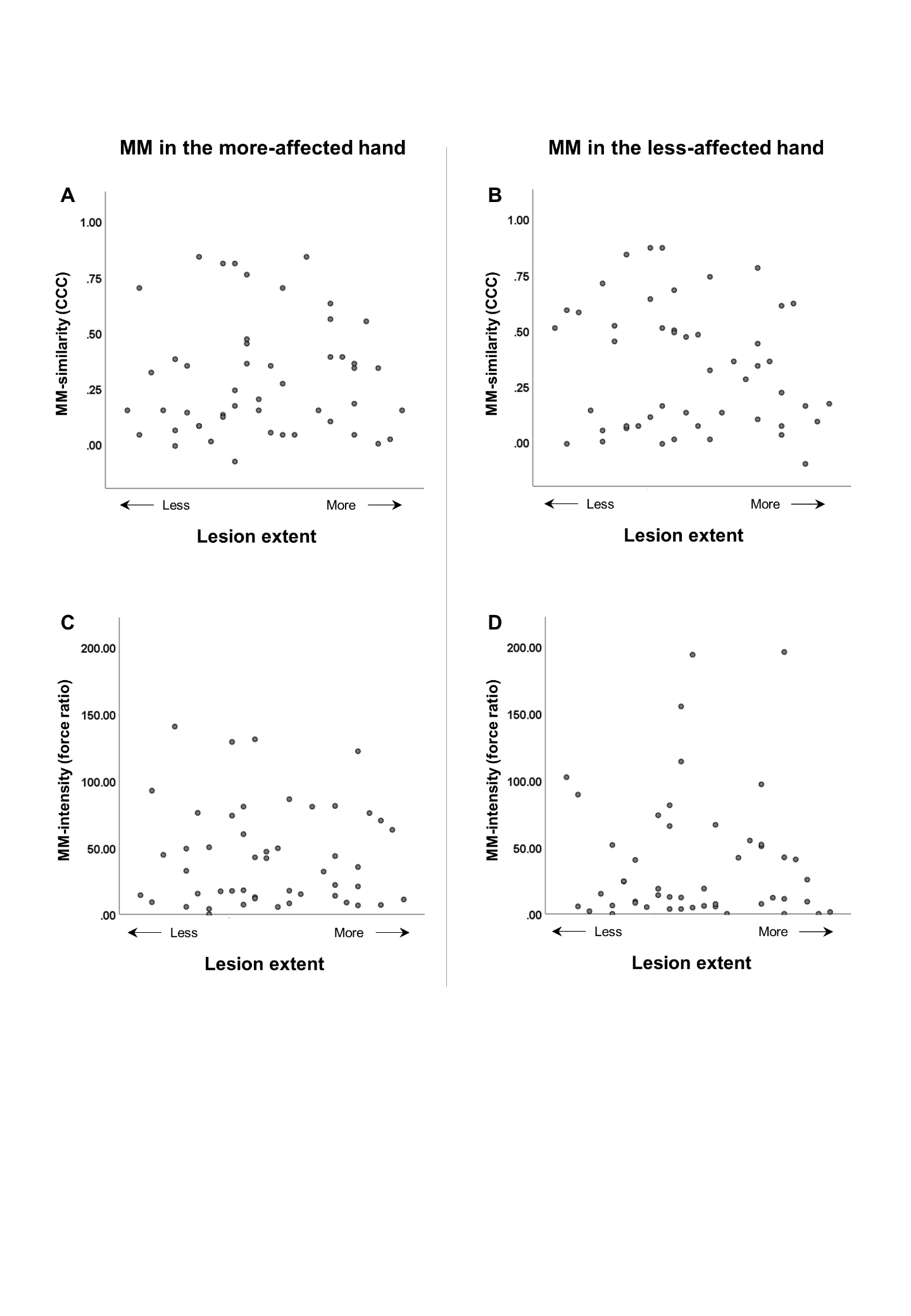
**

**Figure S8**. Individual data points of MM-similarity (A and B) and MM-intensity (C and D) for both the more-affected (left panel) and the less-affected hand (right panel) in relation with the total extent of the lesion.
